# Acid Sphingomyelinase Regulates AdipoRon-Induced Differentiation of Arterial Smooth Muscle Cells via TFEB Activation

**DOI:** 10.3390/ijms26052147

**Published:** 2025-02-27

**Authors:** Xiang Li, Wei Zhao, Zhengchao Wang, Alexandra K. Moura, Kiana Roudbari, Rui Zuo, Jenny Z. Hu, Yun-Ting Wang, Pin-Lan Li, Yang Zhang

**Affiliations:** 1 Department of Pharmacological and Pharmaceutical Sciences, College of Pharmacy, University of Houston, Houston, TX 77204, USA; xli61@central.uh.edu (X.L.); wzhao8@central.uh.edu (W.Z.); akmoura@cougarnet.uh.edu (A.K.M.); kroudbar@cougarnet.uh.edu (K.R.); rzuo@cougarnet.uh.edu (R.Z.); jzhu31@cougarnet.uh.edu (J.Z.H.); ywang264@central.uh.edu (Y.-T.W.); 2 Provincial Key Laboratory for Developmental Biology and Neurosciences, College of Life Sciences, Fujian Normal University, Fuzhou 350007, China; 3 Department of Pharmacology and Toxicology, School of Medicine, Virginia Commonwealth University, Richmond, VA 23298, USA; pin-lan.li@vcuhealth.org

**Keywords:** acid sphingomyelinase, adipoRon, transcription factor EB, autophagy, smooth muscle cell

## Abstract

AdipoRon is a selective adiponectin receptor agonist that inhibits vascular remodeling by promoting the differentiation of arterial smooth muscle cells (SMCs). Our recent studies have demonstrated that activation of TFEB and its downstream autophagy–lysosomal signaling contribute to adipoRon-induced differentiation of SMCs. The present study was designed to examine whether acid sphingomyelinase (ASM; gene symbol *Smpd1*) is involved in mediating adipoRon-induced activation of TFEB–autophagy signaling and inhibition of proliferation/migration in arterial SMCs. Our results showed that adipoRon induced ASM expression and ceramide production in *Smpd1^+/+^* SMCs, which were abolished in *Smpd1^−/−^* SMCs. Compared to *Smpd1^+/+^* SMCs, *Smpd1^−/−^* SMCs exhibited less TFEB nuclear translocation and activation of autophagy signaling induced by adipoRon stimulation. SMC differentiation was further characterized by retarded wound healing, reduced proliferation, F-actin reorganization, and MMP downregulation. The results showed that *Smpd1^−/−^* SMCs were less responsive to adipoRon-induced differentiation than *Smpd1^+/+^* SMCs. Mechanistically, adipoRon increased the expression of protein phosphatases such as calcineurin and PP2A in *Smpd1^+/+^* SMCs. The calcineurin inhibitor FK506/cyclosporin A or PP2A inhibitor okadaic acid significantly attenuated adipoRon-induced activation of TFEB–autophagy signaling. In addition, adipoRon-induced expressions of calcineurin and PP2A were not observed in *Smpd1^−/−^* SMCs. However, activation of calcineurin by lysosomal TRPML1-Ca^2+^ channel agonist ML-SA1 rescued the activation of TFEB–autophagy signaling and the effects of adipoRon on cell differentiation in *Smpd1^−/−^* SMCs. Taken together, these data suggested that ASM regulates adipoRon-induced SMC differentiation through TFEB activation. This study provided novel mechanistic insights into the therapeutic effects of adipoRon on TFEB signaling and pathological vascular remodeling.

## 1. Introduction

Vascular smooth muscle cells (SMCs) are the main cell type in the medial layer of healthy arteries, mainly in a quiescent state and highly differentiated [1]. Vascular SMCs are essential for maintaining vascular integrity and homeostasis, and they are undoubtedly involved in physiological and pathological vascular remodeling [2,3,4]. An increasing number of studies have shown that the aberrant proliferation and migration of vascular SMCs play a key role in the development of cardiovascular diseases (CVDs), such as atherosclerosis [5], inflammation [6], restenosis [7], and aortic aneurysm [8]. Under these pathophysiological conditions, SMCs dedifferentiate from a contractile phenotype to a synthetic phenotype, resulting in enhanced proliferation and migration into the intima, thereby promoting neointimal formation [9]. Hence, it is necessary to develop effective therapeutic approaches to inhibit SMC phenotypic dedifferentiation and explore its potential regulatory mechanisms.

AdipoRon is a selective adiponectin receptor agonist that is widely suitable for clinical research and research that uses it to mimic the beneficial effects of adiponectin [10]. For example, adipoRon may improve insulin resistance and lipotoxicity in type 2 diabetic mice by activating the AdipoR1/2 signaling pathway [11,12]. AdipoRon exerts an anti-apoptotic effect during cardiac ischemia/reperfusion injury by partially activating AMPK [13]. AdipoRon not only exhibits anti-atherosclerotic and anti-inflammatory effects through inhibiting ERK and p38 MAPK activation to reduce the proliferation/migration of vascular SMCs and the levels of pro-inflammatory factors [14] but also suppresses vascular SMC proliferation and neointimal hyperplasia by inhibiting mTORC1/p70S6K signaling [15]. Notably, our recent studies showed that adipoRon promotes SMC differentiation by activating transcription factor EB (TFEB), a master transcription regulator of autophagy–lysosomal signaling [16]. TFEB activation leads to its nuclear translocation and binding to coordinated lysosomal expression and regulation (CLEAR) elements of autophagic genes, including microtubule-associated protein light chain 3 (LC3), ubiquitin-binding protein p62/sequestosome 1 (p62/SQSTM1), and lysosomal-associated membrane protein 1 (LAMP-1). This activation of TFEB–autophagy signaling has been shown to contribute to the inhibitory effects of adipoRon on vascular SMC proliferation and migration [16]. Further studies have found that adipoRon-induced TFEB activation in SMCs was mainly dependent on intracellular calcium ions but not on kinases such as AMPK, ERK1/2, Akt, and mTORC1 [16]. However, the specific mechanism of adipoRon-induced TFEB activation and how intracellular calcium ions are involved in this process remain unclear.

Acid sphingomyelinase (ASM; gene symbol *Smpd1*) is a lysosomal hydrolase that metabolizes sphingomyelin to ceramide and phosphorylcholine, preferentially at an acidic pH [17]. A genetic defect in ASM leads to the accumulation of sphingomyelin and a lysosomal storage disorder named Niemann–Pick disease. Our previous studies have demonstrated a protective role of ASM in maintaining SMC homeostasis by controlling autophagy signaling [18,19]. It was found that ASM deficiency impaired autophagic flux by preventing TRPML1-Ca^2+^-dependent lysosome trafficking and its fusion with autophagosome to form autophagolysosomes [18,20]. Impaired autophagic flux was also associated with increased SMC proliferation and their transformation to a myofibroblast-like phenotype [18,19]. Interestingly, a recent study has shown that inhibition of ASM activates TFEB in endothelial cells [21]. The present study aimed to explore the role of ASM in adipoRon-induced TFEB–autophagy signaling in SMCs and the associated regulatory mechanisms. Therefore, we first investigated the effects of ASM deficiency by genetic ablation of *Smpd1* on adipoRon-induced TFEB activation in primary cultured arterial SMCs, as well as the associated inhibition of SMC proliferation and migration. Moreover, we tested the role of Ca^2+^-dependent phosphatase calcineurin and ceramide-activated protein phosphatase 2A (PP2A) in adipoRon-induced activation of TFEB and inhibition of SMC proliferation and migration. We also examined whether TRPML1-Ca^2+^ channel activation could rescue the beneficial effects of adipoRon in ASM-deficient SMCs. The findings from this study provide novel mechanistic insights into the therapeutic effects of adipoRon on TFEB signaling and pathological vascular remodeling.

## 2. Results

### 2.1. AdipoRon Induces ASM-Mediated Ceramide Signaling in SMCs

**Figure 1:**
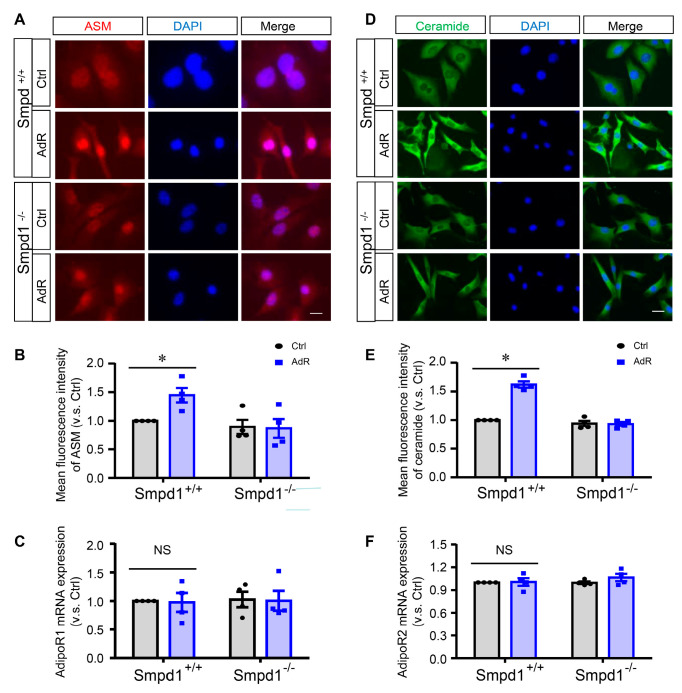
AdipoRon induces ASM-mediated ceramide signaling in SMCs. *Smpd1*^+/+^ and *Smpd1*^−/−^ SMCs were cultured with or without 50 μM adipoRon for 24 h. (**A**): ASM expression shown in the immunofluorescence images. (**B**): Fluorescence intensity of ASM quantified. (**C**): AdipoR1 mRNA expressions. (**D**): Ceramide expression shown in the immunofluorescence images. (**E**): Fluorescence intensity of ceramide quantified. (**F**): AdipoR2 mRNA expressions. * *p* < 0.05 vs. *Smpd1*^+/+^ SMCs without adipoRon. n = 4. Bar = 10 μm. NS: not significant. Ctrl: control. AdR: adipoRon. AdipoR1/2: adipoRon receptor 1/2.

Previous studies indicate that adipoRon promotes SMC differentiation through TFEB activation [15,16]. Here, we investigated the mediating role of ASM in the effects of adipoRon on SMCs. *Smpd1*^+/+^ and *Smpd1*^−/−^ SMCs were treated with or without adipoRon (50 μM) for 24 h, and changes in ASM expression and ceramide production were examined. As shown in Figure 1A,B, immunofluorescent results showed that adipoRon significantly increased the protein expression of ASM in *Smpd1*^+/+^ SMCs but not in *Smpd1*^−/−^ SMCs. Consistently, ceramide production was significantly increased in *Smpd1*^+/+^ SMCs treated with adipoRon, while no significant changes were observed in *Smpd1*^−/−^ SMCs (Figure 1D,E). Additionally, we examined adipoRon receptor 1 (AdipoR1, Figure 1C) and AdipoR2 (Figure 1F) expressions and found no significant changes in *Smpd1*^+/+^ and *Smpd1*^−/−^ SMCs after adipoRon treatment. Together, these results indicate that adipoRon regulates the expression of ASM and ceramide production in arterial SMCs, while *Smpd1* gene ablation can eliminate this regulation.

### 2.2. ASM Deficiency Inhibits adipoRon-Induced TFEB Activation and Autophagy in SMCs

**Figure 2:**
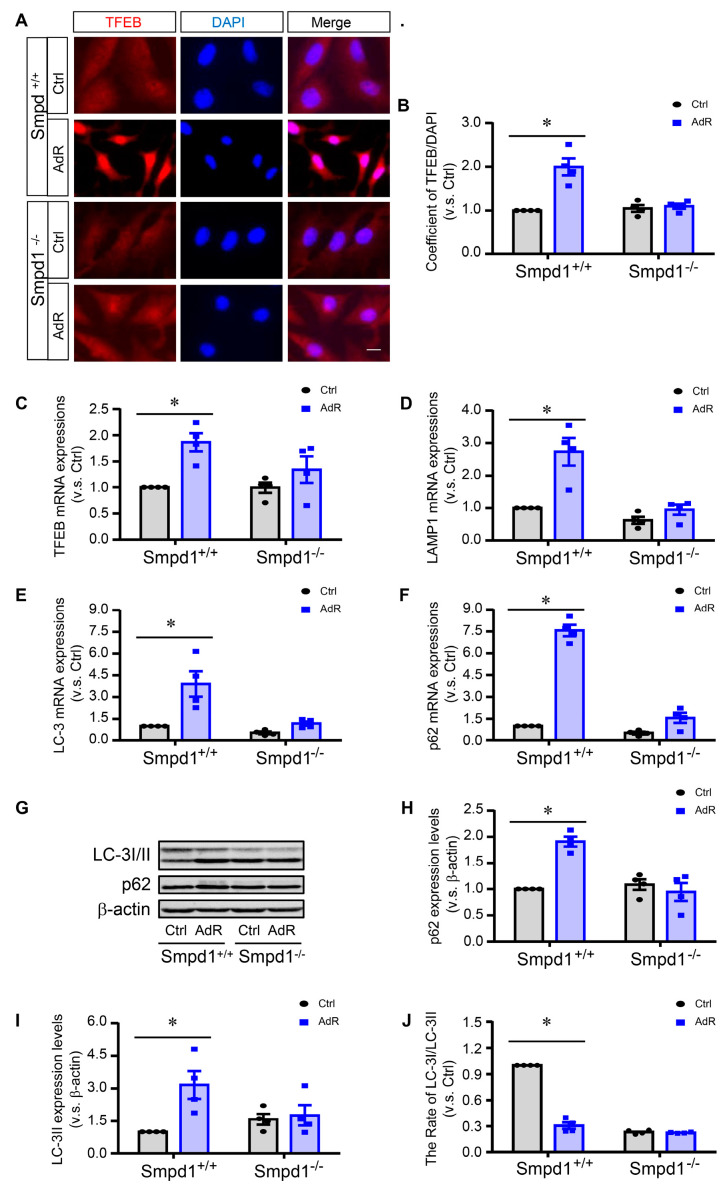
ASM deficiency inhibits adipoRon-induced TFEB activation and autophagy in SMCs. *Smpd1*^+/+^ and *Smpd1*^−/−^ SMCs were cultured with or without 50 μM adipoRon for 24 h. (**A**): TFEB activation shown in the immunofluorescence images (red: TFEB; blue: nuclei). (**B**): TFEB nuclear translocation quantified. (**C**): TFEB mRNA expression. (**D**): LAMP1 mRNA expression. (**E**): LC-3 mRNA expression. (**F**): p62 mRNA expression. (**G**): Immunoblotting images of LC-3I/II and p62. (**H**): p62 expression level quantified. (**I**): LC-3II expression quantified. (**J**): The rate of LC-3I to LC-3II. * *p* < 0.05 vs. *Smpd1*^+/+^ SMCs without AdipoRon. n = 4. Bar = 10 μm. Ctrl: control. AdR: adipoRon.

Next, we investigated the role of ASM in adipoRon-induced TFEB activation and autophagy. We first examined the effect of adipoRon on TFEB nuclear translocation, a key event in TFEB activation, in *Smpd1*^+/+^ and *Smpd1*^−/−^ SMCs. As shown in Figure 2A,B, immunofluorescence studies showed that adipoRon significantly increased the nuclear translocation of TFEB in *Smpd1*^+/+^ SMCs, whereas this TFEB nuclear translocation was absent in *Smpd1*^−/−^ SMCs. We further analyzed the mRNA levels of TFEB and its downstream autophagy and lysosomal genes. The results showed that adipoRon significantly increased the mRNA levels of TFEB (Figure 2C) and its target genes, including LC3 (Figure 2E), LAMP1 (Figure 2D), and p62 (Figure 2F) in *Smpd1*^+/+^ SMCs but not in *Smpd1*^−/−^ SMCs. Consistent with the increase in mRNA levels, adipoRon significantly increased the protein expression of LC3-II and p62, which were inhibited in *Smpd1*^−/−^ SMCs (Figure 2G–J). Together, these results suggest that ASM activity is required for adipoRon-induced TFEB activation and autophagy signaling in arterial SMC.

### 2.3. ASM Deficiency Prevents the Inhibitory Effects of adipoRon on SMC Proliferation and Migration

**Figure 3:**
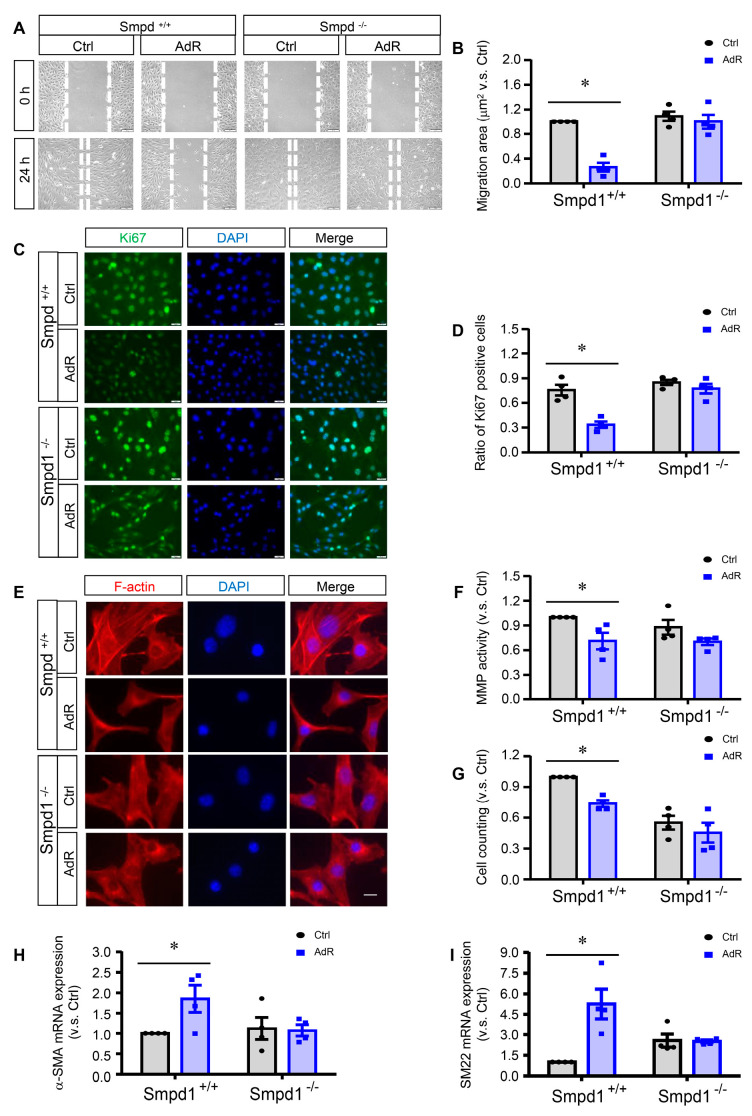
ASM deficiency prevents adipoRon-induced inhibition of proliferation and migration in SMCs. *Smpd1*^+/+^ and *Smpd1*^−/−^ SMCs were cultured with or without 50 μM adipoRon for 24 h. (**A**): The images of scratch assay in *Smpd1*^+/+^ and *Smpd1*^−/−^ SMCs treated with AdR. (**B**): The scratch assay quantified in *Smpd1*^+/+^ and *Smpd1*^−/−^ SMCs treated with AdR. (**C**): Ki67 immunofluorescence images (green) in *Smpd1*^+/+^ and *Smpd1*^−/−^ SMCs treated with AdR. (**D**): Ki67-positive cells quantified in *Smpd1*^+/+^ and *Smpd1*^−/−^ SMCs treated with AdR. (**E**): F-actin immunofluorescence images in *Smpd1*^+/+^ and *Smpd1*^−/−^ SMCs treated with AdR. (**F**): MMP activity quantified in *Smpd1*^+/+^ and *Smpd1*^−/−^ SMCs treated with AdR. (**G**): Cell number counted in *Smpd1*^+/+^ and *Smpd1*^−/−^ SMCs treated with AdR. (**H**): α-SMA mRNA expressions in *Smpd1*^+/+^ and *Smpd1*^−/−^ SMCs treated with AdR. (**I**): SM22 mRNA expressions in *Smpd1*^+/+^ and *Smpd1*^−/−^ SMCs treated with AdR. * *p* < 0.05 vs. the Ctrl as indicated. n = 4. Bar = 10 μm. MMP: matrix metalloproteinase-9. Ctrl: control. AdR: adipoRon. α-SMA: alpha smooth muscle actin. SM22: transgelin (TAGLN).

Migration was first assessed by scratch assay in adipoRon-treated *Smpd1*^+/+^ and *Smpd1*^−/−^ SMCs. As shown in Figure 3A,B, adipoRon significantly decreased cell migration in *Smpd1*^+/+^ SMCs, whereas this decrease was abolished in *Smpd1*^−/−^ SMCs. MMPs are peptidase enzymes involved in extracellular matrix degradation contributing to cell migration. AdipoRon inhibited MMP activity in *Smpd1*^+/+^ SMCs, but not in *Smpd1*^−/−^ SMCs (Figure 3F), demonstrating ASM deficiency prevents adipoRon-induced inhibition of the migration in SMCs. The remodeling of F-actin, a filamentous actin in the cytoskeleton, is a marker event associated with cell migration. Both *Smpd1*^+/+^ and *Smpd1*^−/−^ SMCs without adipoRon treatment exhibited a migratory phenotype with disassembled distribution and aggregation around the perinuclear region of actin filaments without clear filamentous organization (Figure 3E). However, *Smpd1*^+/+^ SMCs treated with adipoRon showed a spindle-like shape and organization of the actin filaments (Figure 3E). This adipoRon-induced F-actin reorganization was not observed in *Smpd1*^−/−^ SMCs (Figure 3E).

The anti-proliferative effect of adipoRon on SMCs was further examined by the immunostaining of proliferation marker Ki67 and by cell counting. As shown in Figure 3C,D, adipoRon significantly decreased the expression of Ki67 in *Smpd1*^+/+^ SMCs, which was not observed in *Smpd1*^−/−^ SMCs. Consistently, adipoRon significantly arrested the cell growth of reduced cell numbers in *Smpd1*^+/+^ SMCs but had no further effect on *Smpd1*^−/−^ SMCs (Figure 3G).

Additionally, the expression levels of SMC differentiation marker alpha-SMA (Figure 3H) and SM22 (Figure 3I) were also examined, and it was found that adipoRon significantly increased their expressions in *Smpd1^+/+^* SMCs but not in *Smpd1^−/−^* SMCs.

### 2.4. Effect of Calcineurin Inhibition on adipoRon-Induced TFEB–Autophagy in SMCs

**Figure 4:**
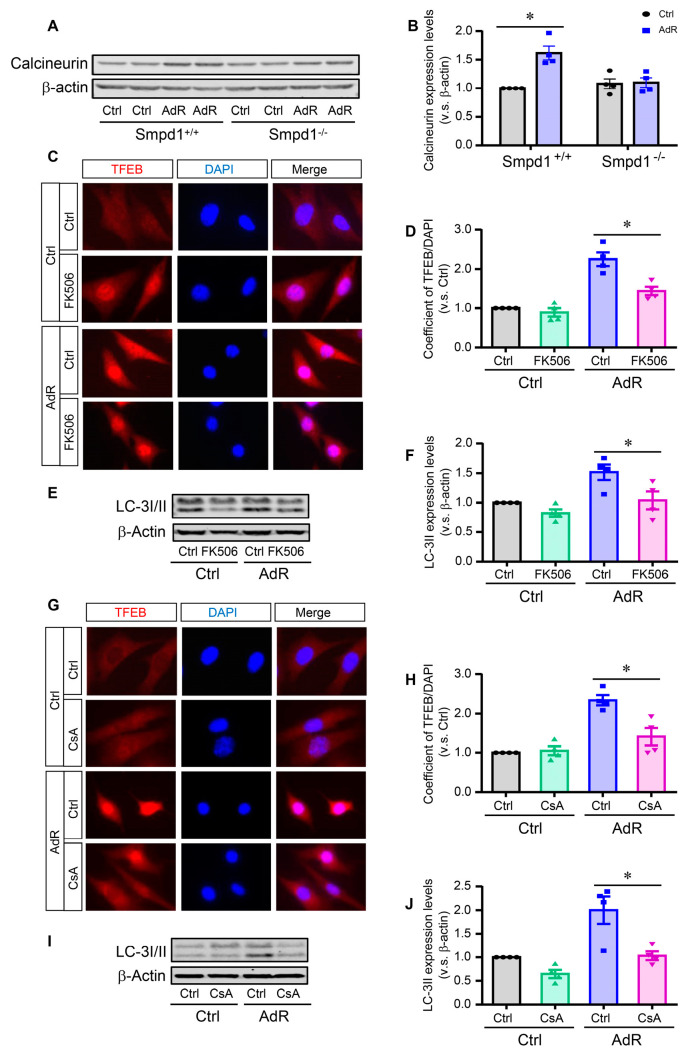
Effect of calcineurin inhibition on adipoRon-induced TFEB activation and autophagy in SMCs. *Smpd1*^+/+^ and *Smpd1*^−/−^ SMCs were cultured with or without 50 μM adipoRon for 24 h. *Smpd1*^+/+^ SMCs were pre-treated with calcineurin inhibitor FK506 or CsA for 1 h before adipoRon treatment. (**A**): Immunoblotting images of calcineurin in *Smpd1*^+/+^ and *Smpd1*^−/−^ SMCs treated with adipoRon. (**B**): Calcineurin expression quantified in *Smpd1*^+/+^ and *Smpd1*^−/−^ SMCs treated with adipoRon. (**C**): TFEB activation shown in immunofluorescence images in *Smpd1*^+/+^ SMCs treated with AdR and FK506 (red: TFEB; blue: nuclei). (**D**): TFEB nuclear translocation quantified in *Smpd1*^+/+^ SMCs treated with AdR and FK506. (**E**): Immunoblotting images of LC-3I/II in *Smpd1*^+/+^ SMCs treated with AdR and FK506. (**F**): LC-3II expression quantified in *Smpd1*^+/+^ SMCs treated with AdR and FK506. (**G**): TFEB activation shown in the immunofluorescence images in *Smpd1*^+/+^ SMCs treated with AdR and CsA (red: TFEB; blue: nuclei). (**H**): TFEB nuclear translocation quantified in *Smpd1*^+/+^ SMCs treated with AdR and CsA. (**I**): Immunoblotting images of LC-3I/II in *Smpd1*^+/+^ SMCs treated with AdR and CsA. (**J**): LC-3II expression quantified in *Smpd1*^+/+^ SMCs treated with AdR and CsA. * *p* < 0.05 vs. the Ctrl as indicated. n = 4. Bar = 10 μm. Ctrl: control. AdR: adipoRon. CsA: cyclosporin A.

Our previous studies have shown that adipoRon-induced TFEB activation is dependent on intracellular Ca^2+^ [11]. As shown in Figure 4A,B, immunoblotting results showed that adipoRon significantly increased the expression of the Ca^2+^-dependent phosphatase calcineurin in *Smpd1*^+/+^ SMCs but not in *Smpd1*^−/−^ SMCs. We then investigated the effects of two calcineurin inhibitors, FK506 and cyclosporin A, on adipoRon-induced TFEB nuclear translocation and LC3 expression in *Smpd1*^+/+^ SMCs. Immunofluorescence studies showed that FK506 (Figure 4C,D) or cyclosporin A (Figure 4G,H) significantly attenuated adipoRon-induced TFEB nuclear translocation. Similarly, FK506 (Figure 4E,F) or cyclosporin A (Figure 4I,J) inhibited adipoRon-induced LC-3II expression. Together, these data suggest that calcineurin acts as a downstream effector of the ASM–ceramide pathway to activate TFEB–autophagy signaling in SMCs.

### 2.5. Effect of PP2A Inhibition on adipoRon-Induced TFEB–Autophagy in SMCs

**Figure 5:**
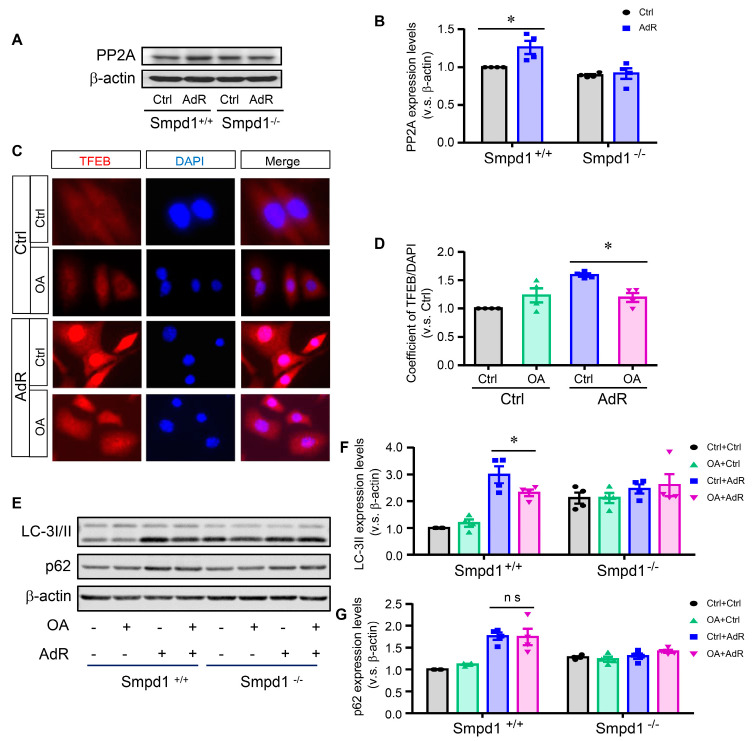
Effect of PP2A inhibition on adipoRon-induced TFEB activation and autophagy in SMCs. *Smpd1*^+/+^ and *Smpd1*^−/−^ SMCs were cultured with or without 50 μM adipoRon for 24 h. *Smpd1*^+/+^ SMCs were pre-treated with PP2A inhibitor OA for 1 h before adipoRon treatment. (**A**): Immunoblotting images of PP2A in *Smpd1*^+/+^ and *Smpd1*^−/−^ SMCs treated with adipoRon. (**B**): PP2A expression quantified in *Smpd1*^+/+^ SMCs treated with AdR and OA. (**C**): TFEB activation showed in the immunofluorescence images in *Smpd1*^+/+^ SMCs treated with AdR and OA (red: TFEB; blue: nuclei). (**D**): TFEB nuclear translocation quantified in *Smpd1*^+/+^ SMCs treated with AdR and OA. (**E**): Immunoblotting images of LC-3I/II and p62 in *Smpd1*^+/+^ and *Smpd1*^−/−^ SMCs treated with AdR and OA. (**F**): LC-3II expression quantified in *Smpd1*^+/+^ and *Smpd1*^−/−^ SMCs treated with AdR and OA. (**G**): p62 expression quantified in *Smpd1*^+/+^ and *Smpd1*^−/−^ SMCs treated with AdR and OA. * *p* < 0.05 vs. the Ctrl as indicated. n = 4. Bar = 10 μm. ns: not significant. PP2A: phosphatase 2A. Ctrl: control. AdR: AdipoRon. OA: okadaic acid.

Ceramide has been shown to exert an anti-proliferative effect through PP2A activation [22]. Interestingly, recent studies have reported that PP2A can dephosphorylate TFEB and mediate oxidative-stress-induced TFEB activation in epithelial cells [23]. We sought to investigate whether PP2A plays a role in adipoRon-induced TFEB activation in SMCs. As shown in Figure 5A,B, adipoRon significantly induced PP2A expression in *Smpd1*^+/+^ SMCs but not in *Smpd1*^−/−^ SMCs. Furthermore, inhibition of PP2A by okadaic acid significantly attenuated adipoRon-induced TFEB nuclear translocation (Figure 5C,D) and LC-3II expression in *Smpd1*^+/+^ SMCs (Figure 5E,F). Notably, okadaic acid had no effect on the protein expression of p62 in SMCs (Figure 5E,G). Together, these data indicate that activation of PP2A may also contribute to adipoRon-induced TFEB activation and autophagy in SMCs.

### 2.6. Lysosomal Ca^2+^ Release by ML-SA1 Rescues adipoRon-Induced Activation of Calcineurin and TFEB in Smpd1^−/−^ SMCs

**Figure 6:**
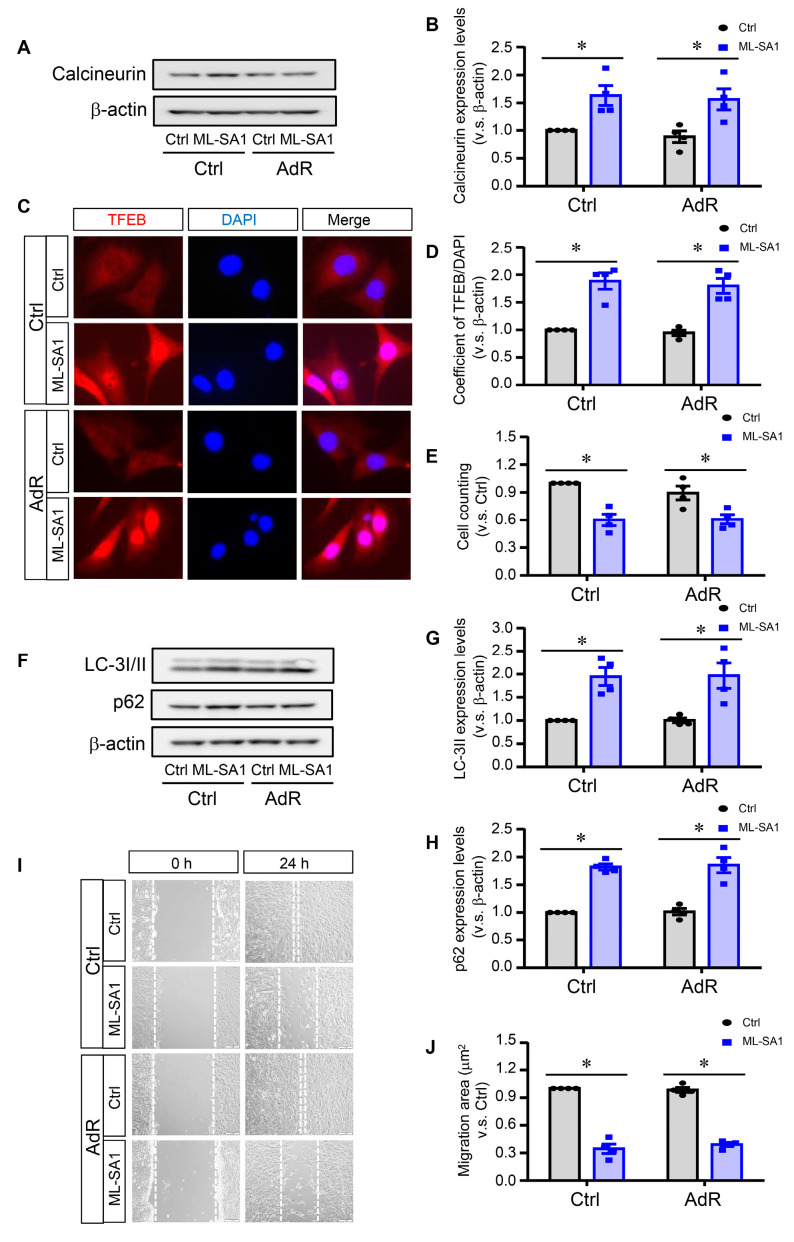
Lysosomal Ca^2+^ release by ML-SA1 rescues adipoRon-induced activation of calcineurin and TFEB in *Smpd1*^−/−^ SMCs. *Smpd1^−/−^* SMCs were pre-treated with ML-SA1 for 1h and then treated with adipoRon for 24 h. (**A**): Immunoblotting images of calcineurin in *Smpd1*^−/−^ SMCs treated with adipoRon and ML-SA1. (**B**): Calcineurin expression quantified in *Smpd1*^−/−^ SMCs treated with adipoRon and ML-SA1. (**C**): TFEB activation shown in the immunofluorescence images in *Smpd1*^−/−^ SMCs treated with adipoRon and ML-SA1 (red: TFEB; blue: nuclei). (**D**): TFEB nuclear translocation quantified in *Smpd1*^−/−^ SMCs treated with adipoRon and ML-SA1. (**E**): Cell number counted in *Smpd1*^−/−^ SMCs treated with adipoRon and ML-SA1. (**F**): Immunoblotting images of LC-3I/II and p62 in *Smpd1*^−/−^ SMCs treated with adipoRon and ML-SA1. (**G**): LC-3II expression quantified in *Smpd1*^−/−^ SMCs treated with adipoRon and ML-SA1. (**H**): p62 expression quantified in *Smpd1*^−/−^ SMCs treated with adipoRon and ML-SA1. (**I**): The images of scratch assay in *Smpd1*^−/−^ SMCs treated with adipoRon and ML-SA1. (**J**): The scratch assay quantified in *Smpd1*^−/−^ SMCs treated with adipoRon and ML-SA1. * *p* < 0.05 vs. the Ctrl as indicated. n = 4. Bar = 10 μm. Ctrl: control. AdR: adipoRon. ML-SA1: lysosomal TRPML1 channel agonist.

Lysosomal TRPML1-Ca^2+^ release has been shown to activate the calcineurin–TFEB signaling axis [24]. Here, *Smpd1*^−/−^ SMCs were treated with a TRPML1 channel activator, ML-SA1, and we examined its impact on adipoRon-induced effects. As shown in Figure 6A,B, ML-SA1 significantly induced calcineurin expressions in *Smpd1*^−/−^ SMCs to a similar level in the presence or absence of adipoRon. Consistently, ML-SA1 significantly increased TFEB nuclear translocation (Figure 6C,D) and protein expression of LC3-II and p62 (Figure 6F–H) in *Smpd1*^−/−^ SMCs treated with or without adipoRon. Furthermore, wound scratch assay showed that ML-SA1 significantly decreased cell migration (Figure 6I,J) and inhibited cell proliferation (Figure 6E) in *Smpd1*^−/−^ SMCs treated with or without adipoRon. These results suggest that ML-SA1 activates calcineurin–TFEB–autophagy signaling and promotes differentiation in *Smpd1*^−/−^ SMCs regardless of adipoRon treatment.

## 3. Discussion

**Figure 7:**
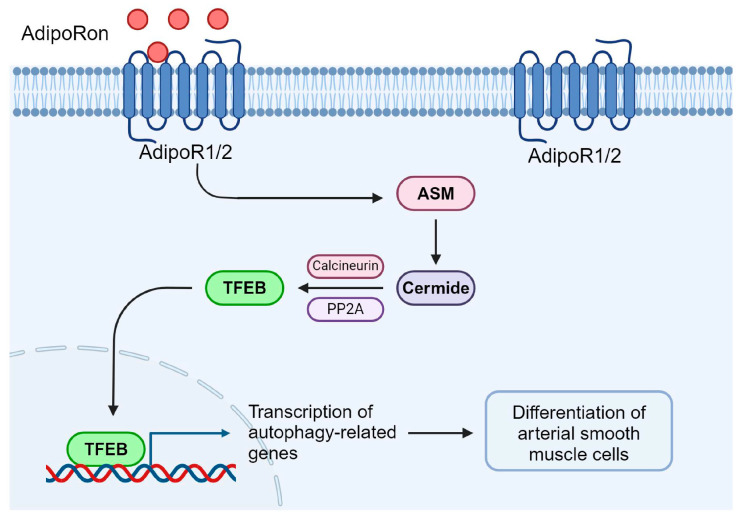
Graphical representation of the mechanism that acid sphingomyelinase regulates adipoRon-induced differentiation of arterial smooth muscle cells via TFEB activation. (Created in BioRender.com).

The aim of this study was to determine the role of ASM in regulating adipoRon-induced TFEB–autophagy signaling and SMC differentiation. Our studies demonstrated that ASM deficiency by genetic ablation of the *Smpd1* gene abolished adipoRon-induced TFEB–autophagy signaling and prevented its inhibitory effects on SMC proliferation and migration. The role of ASM in these adipoRon effects is associated with the upregulation of protein phosphatase calcineurin and PP2A. The lysosomal TRPML1-Ca^2+^ channel agonist ML-SA1 effectively activated calcineurin and TFEB–autophagy signaling in *Smpd1*^−/−^ SMCs and rescued the inhibitory effects of adipoRon on the proliferation and migration of these cells. These results suggest that ASM regulates adipoRon-induced TFEB–autophagy signaling and SMC differentiation through activating protein phosphatases such as calcineurin and PP2A (Figure 7).

Obesity is closely associated with CVD mortality, with more than two-thirds of obese patients dying of CVDs [25]. The high risk of CVD in obese patients is due to changes in cardiac and vascular structure and function [26]. Adipokines are bioactive substances produced by adipose tissue [27]. One of the main reasons for CVD development in obese patients is adipokine disorder, which results in insufficient production of beneficial adipokines or excessive production of deleterious adipokines. Adipokines can regulate the phenotype of SMCs between differentiated and dedifferentiated states upon various stimulations [28]. Adiponectin is a major plasma adipokine that has beneficial effects on CVDs. However, the short half-life and large molecular weight of adiponectin limit its clinical application [16]. AdipoRon is an active synthetic agonist of adiponectin receptor 1/2 (AdipoR1/2) that mimics the biological effects of adiponectin [11]. Recent studies have shown that adipoRon induces SMC differentiation, inhibits SMC proliferation and migration, and attenuates neointima formation in femoral arteries in mice [15,16]. However, in vascular SMCs, the downstream effectors that mediate the adipoRon agonism of AdipoR1/2 remain poorly defined. In the present study, we demonstrated for the first time that adipoRon increased the expression of ASM and ceramide levels in *Smpd1*^+/+^ SMCs, and these adipoRon-induced changes were abolished in *Smpd1*^−/−^ SMCs. Therefore, our results suggest that adipoRon activates the ASM–ceramide signaling pathway in vascular SMCs.

AdipoRon activates downstream signaling pathways by binding to AdipoR1 and AdipoR2, which are essential for the differentiation of SMCs [29,30]. Our present result demonstrates that the mRNA expression levels of AdipoR1 and AdipoR2 are comparable across all groups, indicating that both receptors are expressed in SMCs. The present study did not attempt to identify the specific receptor subtype mediating adipoRon’s effects on SMCs or to delineate the mechanism by which adipoRon–receptor interaction leads to the activation of the ASM–ceramide signaling pathway. Our previous studies have highlighted the pivotal role of intracellular Ca^2+^ in adipoRon-induced TFEB activation in vascular SMCs [16]. Additionally, previous studies have demonstrated that elevated intracellular Ca2+ enhances ASM activation by promoting lysosomal exocytosis [31,32]. Moreover, ASM activation can also occur through reactive oxygen species (ROS) and protein kinase Cδ (PKCδ) [33,34]. It is plausible that these pathways contribute to adipoRon-induced ASM activation in SMCs, which deserve further investigation.

The present study further explored the functional role of ASM in adipoRon-induced TFEB–autophagy signaling in SMCs. Here, we demonstrated that ablation of the *Smpd1* gene in SMCs significantly blocked adipoRon-induced TFEB nuclear translocation and the increase in mRNA or protein levels of TFEB target genes. Notably, *Smpd1*^+/+^ and *Smpd1*^−/−^ SMCs exhibited similar levels of TFEB nuclear translocation, suggesting that ablation of the *Smpd1* gene does not induce TFEB activation at baseline. Consistent with our findings, a recent study has shown that ceramide increases TFEB expression and nuclear translocation and induces lysosomal formation and exocytosis in trophoblast cells [35]. In contrast, our study contradicts a recent study that showed that ASM inhibition with imipramine or SMPD1 siRNA markedly increased TFEB activation in human lung endothelial cells under basal conditions. This effect of ASM inhibition on TFEB activation was further attributed to decreased levels of sphingosine and sphingosine-1-phosphate (S1P) and inhibition of mTOR kinase. Moreover, myriocin, an inhibitor of ceramide de novo synthesis, has been shown to activate TFEB, enhancing fatty acid oxidation and promoting autophagy in airway epithelial cells [36]. The discrepancy between these previous studies and ours regarding the role of ASM/ceramide in TFEB activation or inhibition is unclear. Our previous studies have shown that adipoRon-induced TFEB activation is independent of mTOR kinase inhibition but is rather intracellular Ca^2+^-dependent [16]. Therefore, it is plausible that adipoRon activates ASM–ceramide signaling in SMCs, but it may not affect mTOR kinase activity as it does in endothelial cells. Nonetheless, our results indicate that ASM-mediated ceramide promotes adipoRon-induced TFEB–autophagy in vascular SMCs. Given that the ceramide antibody used in the study detects multiple sphingolipid species, including ceramides (C16 and C24), dihydroceramide, sphingomyelin, and phosphatidylcholine, it is crucial to determine which specific sphingolipid is elevated following adipoRon treatment. Previous studies suggest that adipoRon can modulate sphingolipid pathways, particularly by altering ceramide metabolism [11]. If ASM activity is upregulated, it could lead to increased ceramide production from sphingomyelin [37,38]. Therefore, based on the available data and the role of adipoRon in TFEB activation, an increase in ceramide (particularly C16 and C24 ceramides) is a plausible outcome, potentially contributing to SMC differentiation.

Under various pathological conditions, SMCs dedifferentiate into a more proliferative and migrative state, which causes pathological vascular remodeling and leads to CVDs such as atherosclerosis and restenosis [39,40]. Autophagy is an evolutionarily conserved, repetitive, and dynamic process that degrades and recycles excessive proteins and fragmented organelles through lysosomes [40]. Autophagy plays an important role in cell homeostasis and in maintaining the differentiated state of SMCs by preventing proliferation and migration [41]. The physiological consequences of enhanced autophagy in vascular SMCs have profound implications for cardiovascular function and pathology, particularly in relation to vascular health, arterial remodeling, and cellular stress responses. Autophagy also plays a pivotal role in maintaining vascular SMC homeostasis by regulating survival and function, thereby influencing vascular elasticity, blood pressure, and overall circulatory health [42,43]. During atherosclerosis progression, moderate autophagic activity facilitates the clearance of damaged cells, promoting vascular integrity. However, excessive autophagy may induce vascular SMC death, compromising arterial wall stability and increasing the risk of plaque rupture [43,44,45]. Additionally, heightened autophagy is closely linked to arterial remodeling. Under chronic stress conditions, excessive autophagic activity in vascular SMCs can disrupt cellular function, altering arterial structure and function and promoting atherosclerosis [46]. Autophagy-mediated changes also affect the extracellular matrix composition, further impacting vascular integrity and function [46]. In vascular SMCs, enhanced autophagy plays a crucial role in cellular stress responses. By clearing reactive oxygen species, autophagy mitigates oxidative stress-induced damage [42,43,44]. However, excessive autophagy can increase cellular sensitivity to oxidative stress, ultimately inducing cell death [15,47]. Thus, elucidating the regulatory mechanisms of autophagy in vascular SMCs is essential for developing novel therapeutic strategies to improve cardiovascular disease prognosis.

Genetic or pharmacological activation of the TFEB-mediated autophagy–lysosome system has been shown to reduce atherosclerosis in animal models [48]. Our recent studies have also shown that suppression of TFEB promotes SMC dedifferentiation, while activation of TFEB by trehalose or adipoRon promotes SMC differentiation by inhibiting migration and proliferation [16,41]. In the present study, we demonstrated that ablation of the *Smpd1* gene in SMCs abolished the inhibitory effects of adipoRon on SMC proliferation and migration, MMP downregulation, and F-actin reorganization. These data from previous and present studies support the view that the ASM–TFEB autophagy signaling axis mediates adipoRon-induced SMC differentiation. Our previous studies also showed that ASM promotes autophagic flux by enhancing dynein-dependent lysosomal trafficking and fusion with autophagosomes in SMCs, while ablation of the *Smpd1* gene impairs autophagic flux associated with enhanced SMC dedifferentiation [18]. However, whether the ASM–TFEB axis regulates the adipoRon-induced differentiation by enhancing autophagic flux remains unclear. TFEB has been shown to upregulate lysosomal transmembrane protein TMEM55B, which recruits JIP4 and in turn activates dynein-dependent lysosomal trafficking [49,50]. The ASM-TFEB pathway may coordinate lysosomal trafficking and fusion with autophagosomes to facilitate autophagic flux in vascular SMCs and deserves further investigation.

Recent studies have shown that calcineurin, a Ca^2+^-activated protein phosphatase, is an important activator of TFEB, which dephosphorylates TFEB and triggers its nuclear translocation [24]. We have reported that adipoRon-induced TFEB activation in SMCs is dependent on intracellular Ca^2+^ [11,16]. In the present study, we explored whether calcineurin is involved in adipoRon-induced TFEB activation in SMCs. The results showed that adipoRon increased the expression of calcineurin in *Smpd1*^+/+^ SMCs but not in *Smpd1*^−/−^ SMCs. In addition, inhibition of calcineurin by FK506 or cyclosporin A blocked adipoRon-induced TFEB nuclear translocation and autophagy marker LC3-II expression. Therefore, our data suggest that ASM may control TFEB activation by regulating intracellular Ca^2+^ and thus calcineurin activity. The present study did not further identify how the ASM–ceramide pathway regulates intracellular Ca^2+^ in SMCs. In this regard, several mechanisms have been proposed. First, lysosomal TRPML1-mediated Ca^2+^ release is known to activate calcineurin and TFEB [24,51]. Sphingolipids such as sphingosine and sphingomyelin, but not ceramide, have been reported to regulate TRPML1 channel activity in various mammalian cells, including endothelial cells and podocytes [52,53]. Sphingosine has been reported to enhance TRPML1 channel activity and lysosomal Ca^2+^ release [52], whereas sphingomyelin has the opposite effect [20,54]. It is possible that adipoRon activates ASM–ceramide, thereby increasing lysosomal sphingosine levels through ceramidase-mediated ceramide breakdown and then promoting TRPML1-Ca^2+^ release [55]. Conversely, ASM deficiency or inhibition leads to increased lysosomal sphingomyelin levels, which in turn inhibits TRPML1-Ca^2+^ release. Second, increased ASM–ceramide may lead to increased production of S1P, which binds to membrane G protein-coupled receptors and triggers inositol triphosphate (IP3)-mediated Ca^2+^ release [11]. Third, increased ASM–ceramide may increase cytosolic Ca^2+^ by inhibiting the sarco/endoplasmic reticulum (S/ER) Ca^2+^ ATPase (SERCA) and depleting the S/ER Ca^2+^ pool [56]. Interestingly, inhibition of Ca^2+^ ATPase of SERCA by thapsigargin has been shown to potently activate TFEB in SMCs [16]. Thus, increased ASM–ceramide by adipoRon may inhibit SERCA-dependent Ca^2+^ uptake into the ER lumen, thereby increasing cytosolic Ca^2+^ concentrations. Therefore, ASM–ceramide may have multifactorial effects on intracellular Ca^2+^ that controls calcineurin and TFEB activity and contribute to the effects of adipoRon on SMC homeostasis.

Moreover, this study showed that ceramide-activated protein phosphatase PP2A is also involved in adipoRon-induced TFEB activation in SMCs. It has been well established that ceramide can activate PP2A, thereby exerting antiproliferation effects [19,22,57]. Recent studies have shown that TFEB is activated upon induction of acute oxidative stress by sodium arsenite through an mTORC1-independent but PP2A-dependent process [23]. In the present study, adipoRon increased PP2A expression in SMCs, while *Smpd1* gene ablation suppressed this expression. Furthermore, inhibition of PP2A with okadaic acid significantly reduced TFEB nuclear translocation and attenuated LC-3II expression. These results suggest that the ASM–ceramide–PP2A axis plays a role in adipoRon-induced TFEB activation in SMCs. PP2A can directly dephosphorylate TFEB at several serine residues to facilitate TFEB activation [23]. Instead, PP2A may decrease the activity of the Ca^2+^ ATPase of the SERCA. SERCA activity is regulated by the inhibitory protein phospholamban in many cell types, including SMCs [58]. Phospholamban can be phosphorylated by calmodulin-dependent protein kinase II (CaMKII) or protein kinase A (PKA) [59]. Phospholamban inhibits SERCA activity, and its phosphorylation results in its dissociation from SERCA and release of inhibition [59]. PP2A has been shown to dephosphorylate phospholamban, thereby inhibiting SERCA [59]. Therefore, it is possible that PP2A inhibits SERCA, thereby increasing cytosolic Ca^2+^ and enhancing calcineurin and TFEB activity.

The present study further investigated the effects of TRPML1 channel activation on TFEB–autophagy and proliferation and migration in *Smpd1*^−/−^ SMCs. Previous studies have demonstrated that ML-SA1 as a TRPML1 channel agonist activated lysosomal Ca^2+^ release in vascular SMCs and ECs [40,52,60]. Moreover, ML-SA1 enhanced lysosome trafficking and its fusion with late endosomes/multivesicular bodies in these cells. The Ca^2+^ bursts of lysosomes can activate global Ca^2+^ release from the sarcoplasmic reticulum (SR) to increase cytosolic Ca^2+^, which may be sufficient to drive the dynein-dependent movement of lysosomes along microtubules to encounter other cellular vesicles such as multivesicular bodies or autophagosomes [40]. In the present study, we demonstrated that ML-SA1 induced calcineurin expressions, triggered TFEB nuclear translocation, and increased autophagy signaling in *Smpd1*^−/−^ SMCs. ML-SA1-induced calcineurin–TFEB autophagy signaling in *Smpd1*^−/−^ SMCs was also accompanied by the inhibition of their proliferation and migration. Recently, sphingolipids were found to regulate TRPML1 channel activity [40]. ASM deficiency may increase substrate lysosomal sphingomyelin but reduce lysosomal ceramide or its metabolites sphingosine and S1P. The ceramide metabolite sphingosine was found to directly enhance TRPML1 channel activity, but ceramide had no effect [52,60]. Ablation of the Asah1 gene to inhibit acid ceramidase activity and sphingosine production remarkably decreased ML-SA1-induced Ca^2+^ release through TRPML1 channels [60]. Shen et al. showed that inhibited TRPML1 activity and a reduction in lysosomal Ca^2+^ release observed in Niemann–Pick cells were associated with lysosomal sphingomyelin accumulation [54]. These studies suggest that lysosomal TRPML1-mediated Ca^2+^ release is a major contributor to the global Ca^2+^ increase, thereby activating calcineurin and TFEB autophagy signaling and promoting SMC differentiation. In addition, these studies implicate that ASM/ceramide serves as an upstream regulator of lysosomal Ca^2+^ release in vascular SMCs. Interestingly, the effects of TRPML1 channel activation on TFEB–autophagy and SMC differentiation were observed both at baseline and under adipoRon stimulation. Therefore, these findings support the view that TRPML1 activation by ML-SA1 is sufficient to activate the TFEB–autophagy signaling and induce differentiation in *Smpd1*^−/−^ SMCs, thereby rescuing the beneficial effects of adipoRon on SMC hemostasis in the absence of ASM.

## 4. Materials and Methods

### 4.1. Reagents and Antibodies

Information on primary and secondary antibodies for immunoblotting and immunofluorescence staining is provided in Supplementary Table S1. The following reagents were used: adipoRon (ab144867; Abcam, Cambridge, UK) and Triton X-100 (Sigma, Kawasaki, Japan, X100).

### 4.2. Mice

All experimental protocols were reviewed and approved by the Animal Care Committee of the University of Houston. All animals were kept in a standardized manner in the animal center, University of Houston.

### 4.3. Primary Culture of Arterial SMCs from Mice

Mouse arterial *Smpd1^+/+^* and *Smpd1^−/−^* SMCs were isolated from the corresponding mice as previously described [16,18,19]. Briefly, 6-week-old mice were deeply anesthetized with pentobarbital sodium (ip, 25 mg/kg). Mouse hearts were excised and immersed in an ice-cold Krebs–Henseleit (KH) solution. Then, a 25-gauge needle filled with Hanks’ buffered saline solution (HBSS) was inserted into the heart close to the aortic valve through the aortic lumen and tied when the needle tip reached the base of the heart. An infusion pump was started with a 20 mL syringe containing warm HBSS at a rate of 0.1 mL/min for 15 min. Then, the HBSS was replaced with a warm enzyme solution (containing 1 mg/mL collagenase type 1, 0.5 mg/mL soybean trypsin inhibitor, 3.0% BSA, and 2.0% antibiotics) which was flushed through the heart at a rate of 0.1 mL/min. The outflow perfusion fluid was collected at 30, 60, and 90 min intervals. The heart was cut to open the apex to flush out the cells inside the ventricle after collecting all outflow fluid at 90 min. The flushed cells were centrifuged at 1000 rpm for 10 min, and the pellets were resuspended in advanced Dulbecco’s modified Eagle’s medium (DMEM) with 10% fetal bovine serum (FBS), 10% mouse serum, and 2.0% antibiotics. The isolated cells were plated on 2.0% gelatin-coated plates and incubated in 5.0% CO_2_ at 37 °C. These isolated cells were confirmed as SMCs originated mainly from coronary arteries by positive staining with α-SMA antibodies and the SMC morphology. The culture medium was replaced 3 days after cell isolation and then twice each week until the cells grew to confluency. All studies were performed with cells at passages 5~8. In this study, SMCs were cultured under a dedifferentiation condition in full-serum medium (DMEM with 10% FBS), if not particularly mentioned.

### 4.4. Immunoblotting

These SMCs were treated correspondingly and then collected for the following immunoblotting analysis as described previously [16,19]. Briefly, the cell samples were lysed and denatured first, and 20 μg proteins were separated by SDS-PAGE before transferring onto a PVDF membrane. The membrane was blocked and then incubated with primary antibodies, followed by corresponding secondary antibodies and substrates by using LI-COR Odyssey Fc System.

### 4.5. Real-Time Quantitative PCR

Quantitative RT-PCR was performed as previously described [16,19]. Briefly, the total RNA was extracted by using the Aurum Total RNA Mini Kit (732-6820, Bio-Rad, Hercules, CA, USA), and then reverse transcribed into cDNA by using iScript Reverse Transcription Supermix (1708841, Bio-Rad). Quantitative RT-PCR was conducted by using iTaq Universal SYBR Green Supermix (1725121; Bio-Rad) on the Bio-Rad CFX Connect real-time system with the primers (Supplementary Table S2) according to the manufacturer’s instructions. The expression of β-actin was used as the internal control, and the results were presented by the 2^−ΔΔCt^ method.

### 4.6. Immunofluorescence Staining

Immunofluorescence staining was performed as previously described [16,19]. Briefly, approximately 1.0 × 10^4^ SMCs were cultured and treated with adipoRon for 24 h and then fixed for 15 min by using 4.0% paraformaldehyde at room temperature. Cells were blocked using 5.0% BSA in PBST for 1 h and then incubated with primary antibodies overnight at 4 °C. Secondary fluorescent antibodies and DAPI were incubated according to the corresponding primary antibody for 1 h and then mounted after washing. For phalloidin staining of F-actin in cultured cells, fixed and permeabilized cells were incubated with Alexa Fluor 568-conjugated phalloidin (1:50) for 30 min, washed with PBS, and mounted with an anti-fluorescence quenching agent. Results were visualized by the Olympus IX73 Imaging System and then analyzed with Image-Pro Plus 6.0 software for the Pearson correlation between colocalization efficiency and average fluorescence density.

### 4.7. Wound Scratch Assay

Cell migration was assessed by using a wound scratch assay as previously described [16]. Briefly, 90% of confluent SMCs were starved in low-serum media (0.1% FBS) overnight. Scratch wounds were created using a 2 mm-wide pipette tip. Cells were cultured in full-serum medium (10% FBS) with indicated treatment. After 24 h, the scratched area of cells was imaged using the Olympus IX73 imaging system. The average wounded area was quantified using Image-Pro Plus 6.0 software.

### 4.8. MMP Activity Assay

The activity of MMP was determined by using an MMP Activity Kit (Abcam, ab112146) according to the manufacturer’s instructions.

### 4.9. Statistics Analysis

Data are presented as mean ± SE. Experiment results were analyzed by Student’s *t* test or one/two-way ANOVA by GraphPad Prism 6.0 (GraphPad Software). *p* < 0.05 was considered statistically significant.

## 5. Conclusions

In summary, for the first time, we elucidated the critical regulatory role of ASM in adipoRon-induced TFEB–autophagy signaling and the consequent inhibition of SMC proliferation and migration. Our study provides new mechanistic insights into how ASM controls the TFEB–autophagy signaling by regulating TRMPL1-mediated Ca^2+^ and its implication for adipoRon-induced beneficial effects on SMC homeostasis.

## Data Availability

The original contributions presented in this study are included in the article/Supplementary Material. Further inquiries can be directed to the corresponding author.

## Supplementary Materials

The following supporting information can be downloaded at https://www.mdpi.com/article/10.3390/ijms26052147/s1.
