# Supplementary material for: Acid Sphingomyelinase Regulates AdipoRon-Induced Differentiation of Arterial Smooth Muscle Cells via TFEB Activation
Source: Int J Mol Sci. 2025 Feb 27;26(5):2147. doi: 10.3390/ijms26052147 (PMC11899876; doi:10.3390/ijms26052147)
Supplement: Supplementary file 1 [file ijms-26-02147-s001.zip › ijms-3419955-supplementary.pdf]

Supplementary Table S1

Table S1. The information of primary and secondar antibodies for immunoblotting and immunofluorescence staining.

Antibody	Company	Catalogue Number
TFEB	Bethyl Laboratories	A303-673A
ASM	Novus Biologies	NBP2-45889
Ceramide	Enzo	ALX-804-196-T050
LC-3I/II	Cell Signaling Technology	12741S
p62/SQSTM1	Abcam	ab109012
PP2A	Cell Signaling Technology	2259S
Calcineurin	Cell Signaling Technology	2614S
Ki67	Abcam	ab16667
β-actin	Cell Signaling Technolog	3700S
Donkey anti-mouse IgG (H+L), HRP	Thermo Fisher	A16011
Goat anti-rat IgG-HRP	Thermo Fisher	629520
Stabilized peroxidase-conjugated goat anti-rabbit IgG (H+L)	Invitrogen	32460
IRDye 800CW donkey anti-mouse IgG (H+L)	LICOR	926-32212
IRDye 800CW donkey anti-rabbit IgG (H+L)	LICOR	926-32213
Donkey Alexa Fluor 488 conjugate anti-mouse IgG (H+L)	Thermo Fisher	A-21202
Donkey Alexa Fluor 488 conjugate anti-rabbit IgG (H+L)	Thermo Fisher	A21206
Donkey Alexa Fluor 555 conjugate anti-mouse IgG (H+L)	Thermo Fisher	A-31570
Donkey Alexa Fluor 555 conjugate anti-rabbit IgG (H+L)	Thermo Fisher	A-31572

Supplementary Table S2

Table S2, The primers of quantitative RT-PCR,

Gene	The primer sequences	
TFEB	Forward	5′-CAG CAG GTG GTG AAG CAA GAG T-3′
	Reverse	5′-TCC AGG TGA TGG AAC GGA GAC T-3′
LC-3	Forward	5′-CGT CCT GGA CAA GAC CAA GT-3′
	Reverse	5′-ATT GCT GTC CCG AAT GTC TC-3′
p62	Forward	5′-AGG GAA CAC AGC AAG CT-3′
	Reverse	5′-GCC AAA GTG TCC ATG TTT CA-3′
LAMP-1	Forward	5′-ACA TCA GCC CAA ATG ACA CA-3′
	Reverse	5′-GGC TAG AGC TGG CAT TCA TC-3′
α-SMA	Forward	5′-TCG GAT ACT TCA GCG TCA GGA-3′
	Reverse	5′-GTC CCA GAC ATC AGG GAGTAA-3′
SM22	Forward	5′-ACC CTC CAT GGT CTT CAA GCA GAT-3′
	Reverse	5′-ATC TCC ACG GTA GTG CCC ATC ATT-3′
AdipoR1	Forward	5′-TCC GAA AGG TCC TCC GTA AAC A-3′
	Reverse	5′-CTC CAT CAA CTT CCC GAC CG-3′
AdipoR2	Forward	5′-ACA AGA ATC CGT GGA GCT CAG-3′
	Reverse	5′-GCT GGC TCG TTC ATG GGA TA-3′
β-actin	Forward	5′-TCG CTG CGC TGG TCG TC-3′
	Reverse	5′-GGC CTC GTC ACC CAC ATA GGA-3′
